# Cognitive Impairment and Its Associated Determinants Among the Elderly Population of Telangana, India: An Analytical Prevalence Study

**DOI:** 10.7759/cureus.61535

**Published:** 2024-06-02

**Authors:** Dhanalakshmi V, Vaman Kulkarni, Remya M John, Kartikeyan Nadella, Rashmi Kundapur

**Affiliations:** 1 Community Medicine and Family Medicine, All India Institute of Medical Sciences, Bibinagar, IND

**Keywords:** public health, urban population, india, loneliness, aging, cognitive dysfunction

## Abstract

Introduction: Dementia is an insidious cognitive disorder featuring a decline in cognition that is not well explained by the physiology of aging. Dementia includes a group of disorders that are distinguished by a gradual loss of both cognition and the capability to execute day-to-day functions.

Materials and methods: We conducted a cross-sectional study among 384 elderly participants in areas surrounding the All India Institute of Medical Sciences, Bibinagar, Telangana, India. Those with more than 65 years of age were included in the study, and those suffering from serious illnesses were excluded. The Montreal Cognitive Assessment (MOCA) scale, the University of California and Los Angeles (UCLA) Loneliness Scale, and the Patient Health Questionnaire (PHQ-9) were used to assess cognitive status, loneliness, and depression, respectively, among the study participants. Logistic regression was performed to identify factors associated with cognitive impairment (CI), depression, and loneliness.

Results: The average MOCA score of the study participants was 14.9 ± 6.9, with 28.6% of the participants exhibiting severe CI. Nearly half of the participants (49.2%) experienced moderate to high degrees of loneliness, and 39.3% experienced moderate to severe depression. Important factors found to be associated with severe CI were illiteracy (adjusted odds ratio (AOR): 2.85, 95% CI: 1.35-4.45), urban residence (AOR: 0.18, 95% CI: 0.04-0.81), living with a spouse (AOR: 0.23, 95% CI: 0.11-0.78), not consuming alcohol (AOR: 0.35, 95% CI: 0.14-0.87), and depression (AOR: 4.49, 95% CI: 1.37-14.67).

Conclusion: CI is a serious public health problem in India. With the increasing proportion of the elderly population in the near future, CI levels will increase, especially in countries like India. Timely interventions such as early identification through community-based screening, the inclusion of a geriatric health component in primary health care, and proper counseling will help address this problem at a grassroots level.

## Introduction

Dementia is an insidious cognitive disorder characterized by a decline in cognition that is not well explained by the physiology of aging. According to the World Alzheimer Report 2017, over five crores of people are affected by dementia globally, which is estimated to increase to 15 crores by 2050, particularly among the low and middle-income countries (LMIC) [1]. According to the Longitudinal Ageing Study in India, dementia has a prevalence of 7.4% and 8.3% among the elderly populations of India and Telangana, respectively [2]. Dementia includes a group of disorders that are distinguished by a gradual loss of cognition and the capability to execute day-to-day functions. The disease is associated with neurological and psychiatric symptoms, as well as unwanted behaviors, of different varieties and severity. The basic pathology is usually degeneration related to different variants such as vascular dementia, Alzheimer’s disease dementia, frontotemporal dementia, and dementia with Lewy bodies [3]. Dementia mostly affects those aged 65 years and older, as its degenerative effects occur in old age. Along with these non-modifiable factors, certain modifiable factors, such as illiteracy, raised blood pressure, deafness, smoking, obesity, depression, lack of exercise, diabetes mellitus, lack of social contact, excessive liquor consumption, traumatic brain injury, and air pollution, are also associated with dementia [1].

Depression occurring in those of advanced age is an independent determinant for the development of dementia [4] and is considered a prodromal symptom of the disease [5]. Identification of the clinical features of elderly individuals with depression that are suggestive of nascent dementia would facilitate appropriate categorization of those at high risk for early progression. The pattern and severity of symptoms of depression in the later part of life have surfaced as major factors in assessing the probability of progression to dementia [6]. Hayley proposed that the combination of chronic stress and inflammation compromises vascular and brain function, leading to depression and eventually dementia [7]. Loneliness, which affects social behavior, is also associated with dementia, as those with high degrees of loneliness often have reduced social mobility and poor relationship status. Dementia-related risk is also higher in smokers compared to that of never-smokers, with cessation of smoking for more than three years reducing the risk level to that of never-smokers [8-11].

Being the second most populous nation, India is home to many elderly people. Members of this group are largely uneducated and reside in rural areas, away from urban health facilities, with high rates of undiagnosed dementia. Given the substantial heterogeneity of the Indian population, the burden of dementia among sub-populations may be distributed unequally. However, there is a paucity of literature on dementia-related determinants in these sub-populations. In this work, we assess the burden of dementia, its associated determinants, and the above-mentioned factors, including lifestyle, of the elderly people of Telangana. Our results may help the government to create rural health policies accordingly.

## Materials and methods

We conducted a cross-sectional study on elderly persons living in areas surrounding the All India Institute of Medical Sciences (AIIMS), Bibinagar, Telangana. The study was conducted from January 2023 to December 2023. Those aged ≥ 65 years were included in the study, and persons with serious illnesses were excluded. A sample size of 384 was calculated with N-Master software, considering a power of 80%, a confidence level of 95%, a prevalence of dementia in India of 10%, and a relative precision of 20%. The following formula was used for sample size calculation: N = z2PQ/ε2P. Where N is the sample size, z is a constant (1.96), P is the prevalence, Q is equal to 1-P, and d is the relative precision. Data were collected via purposive sampling.

The Montreal Cognitive Assessment (MOCA) scale was used to determine the range and severity of cognitive impairment (CI) among the study participants. The total score ranges from 0 to 30, with lower scores indicating severe CI and higher levels of dementia. Dementia severity was categorized (as described in the literature) as follows: 24-30, no CI; 20-24, mild CI; 10-20, moderate CI; and ≤10, severe CI [12].

The Patient Health Questionnaire (PHQ-9) was used to assess depressive symptoms among the study participants. The overall score ranges from 1 to 27, with higher scores suggesting severe depression. The scores were categorized as follows: 1-4, minimal depression; 5-9, mild depression; 10-14, moderate depression; 15-19, moderately severe depression; and 20-27, severe depression [13].

The revised University of California Los Angeles (UCLA) Loneliness Scale was used to assess loneliness levels among the study participants. The overall score ranges from 20 to 80, with higher scores associated with a higher degree of loneliness. The scores were categorized as follows: 20-34, low degree of loneliness; 35-49, moderate degree of loneliness; 50-64, moderately high degree of loneliness; and 65-80, high degree of loneliness [14].

The collected data were entered and analyzed using SPSS version 25 (IBM Corp., Armonk, NY). Descriptive variables were reported in terms of percentages, means, and averages. Associations between variables of interest were tested using chi-squared and Fisher’s exact tests and unadjusted odds ratios were calculated. To remove the effect of unknown confounder variables, binary logistic regression tests were conducted. All dependent variables found to be statistically significant in the univariate analysis were subjected to binary logistic regression. The Hosmer-Lemeshow goodness-of-fit test was used to determine whether the logistic regression fit the normal distribution of the data, and p < 0.05 was considered statistically significant. The study protocol was approved by the Institutional Ethics Committee of AIIMS, Bibinagar. All study participants were provided with participant information sheets. Written informed consent to participate was obtained from each participant. All collected information was kept confidential. Those who were found to have serious diseases were provided with referrals.

## Results

Approximately 384 elderly people participated in the study. The mean age (± SD) of the participants was 73.28 ± 7.11 years. More than half of the participants (n = 218, 56.8%) were males, and 166 (43.2%) were females. More than half of the participants (n = 222, 57.8%) had no formal years of schooling, whereas others had varying levels of education, including primary school (n = 48, 12.5%), middle school (n = 32, 8.3%), high school (n = 36, 9.4%), intermediate/diploma (n = 34, 8.9%), graduate (n = 11, 2.9%), and professional qualifications (n = 11, 0.3%). The majority of the participants (n = 201, 52.3%) performed elementary jobs (e.g., watchman, peon, or domestic servant) as their main source of income, and 50 participants (13%) worked craft and related trade jobs. Other occupational categories included plant and machine operators/assemblers (n = 45, 11.7%), skilled agricultural and fishery workers (n = 12, 3.1%), skilled workers in shops and market sales (n = 17, 4.4%), and legislators/senior officials/managers (n = 8, 2.1%). A total of 51 (13.3%) participants were unemployed during the study period. The median (interquartile range, IQR) income of the participants was Rs. 2000 (2000, 8000). The residence type of the participants was evenly distributed between rural (n = 192, 50%) and urban (n = 192, 50%) areas. The majority of the participants (n = 233, 60.7%) were married and lived with their spouses, and 144 (37.5%) were widows or widowers. The majority (n = 169, 44%) of the participants had two children, whereas 27 (7%) did not have any children. More than one-third (n = 135, 35.2%) of the participants had their children residing in the same house as them. Nearly half (n = 180, 46.9%) of the participants were visited by their children at a frequency of one to 10 visits per year, 13 (3.4%) were visited by their children more than 10 times a year, and 49 (12.8%) were not visited by their children in the last year. Nearly all (n = 378, 98.4%) of the participants did not participate in any health insurance schemes. A total of 157 (40.9%) participants were smokers, and 276 (71.9%) consumed alcohol. The sociodemographic characteristics of the participants are summarized in Table 1.

**Table 1:** Sociodemographic characteristics of the study participants (n = 384).

	Variable	n (%)
1	Age ± standard deviation (years)	73.28 ± 7.11
2	Gender	
	Male	218 (56.8)
	Female	166 (43.2)
3	Education	
	Illiterate	222 (57.8)
	Primary	48 (12.5)
	Middle school	32 (8.3)
	High school	36 (9.4)
	Intermediate/diploma	34 (8.9)
	Graduate	11 (2.9)
	Professional	1 (0.3)
4	Occupation	
	Unemployed	51 (13.3)
	Elementary occupation	201 (52.3)
	Plant & machine operators, assemblers	45 (11.7)
	Craft & related trade workers	50 (13.0)
	Skilled agricultural & fishery workers	12 (3.1)
	Skilled workers, shop, and market sales workers	17 (4.4)
	Legislators, senior officials, managers	8 (2.1)
5	Monthly income – median (Q1, Q3)	2000 (2000, 8000)
6	Marital status	
	Married	233 (60.7)
	Widow/widower	144 (37.5)
	Divorced	2 (0.5)
	Single	5 (1.3)
7	Living with spouse	
	Yes	233 (60.7)
	No	151 (39.3)
8	Type of residence area	
	Rural	192 (50.0)
	Urban	192 (50.0)
9	Number of children	
	0	27 (7.0)
	1	60 (15.6)
	2	169 (44.0)
	3	68 (17.7)
	4	43 (11.2)
	5	16 (4.2)
	6	1 (0.3)
10	Frequency of children’s visits to family	
	No visits	49 (12.8)
	1–10 visits	180 (46.9)
	11–20 visits	12 (3.1)
	>20 visits	1 (0.3)
	Dead children	7 (1.8)
	Living in the same house	135 (35.2)
11	Availing health insurance	
	Yes	6 (1.6)
	No	378 (98.4)
12	Smoking	
	Yes	157 (40.9)
	No	227 (59.1)
13	Alcohol consumption	
	Yes	276 (71.9)
	No	108 (28.1)

The mean MOCA score was found to be 14.9 ± 6.9. A total of 110 (28.6%) participants had severe CI, 135 (35.2%) had moderate CI, 107 (27.9%) had mild CI, and only 32 (8.3%) had no CI. Of the participants, 114 (29.7%) exhibited minimal depressive symptoms, 119 (31.0%) exhibited mild depressive symptoms, 92 (24.0%) exhibited moderate depressive symptoms, 36 (9.4%) exhibited a combination of moderate and severe depressive symptoms, and only 23 (6.0%) exhibited severe depressive symptoms. More than half (n = 195, 50.8%) of the participants had low degree of loneliness, 154 (40.1%) had moderate levels of loneliness, 28 (7.3%) had moderate to high levels of loneliness, and seven (1.8%) had severe levels of loneliness.

Higher degrees of CI were found among the following categories: age ≥ 80 years (n = 33, 38.8%), females (n = 62, 37.3%), illiterate (n = 98, 44.1%), rural residence (n = 91, 47.4%), living alone (n = 62, 41.1%), alcohol use (n = 90, 32.6%), higher degrees of depression (n = 102, 37.8%), and higher degrees of loneliness (n = 82, 43.4%). These associations were found to be statistically significant, as seen in Table 2.

**Table 2:** Factors associated with cognitive impairment among the elderly (n = 384).

Factor		Severe cognitive impairment, n (%)	Some cognitive impairment, n (%)	p-value	Unadjusted odds ratio (95% CI)
Age group					
<80 years and ≥80 years		77 (25.8)	222 (74.2)	0.019	1.21 (1.01, 1.45)
		33 (38.8)	52 (61.2)		
Gender					
	Male	48 (22)	170 (78)	<0.001	1.24 (1.08, 1.42)
	Female	62 (37.3)	104 (62.7)		
Educational status					
No formal education		98 (44.1)	124 (55.9)	<0.001	5.95 (3.39, 10.47)
Formal education		12 (7.4)	150 (92.6)		
Occupational status					
Currently not employed		19 (37.3)	32 (62.7)	0.144	1.36 (0.92, 2.02)
Currently employed		91 (27.3)	242 (72.7)		
Type of residence area					
Rural		91 (47.4)	101 (52.6)	<0.001	4.79 (3.05, 7.52)
Urban		19 (9.9)	173 (90.1)		
Living with spouse					
	Yes	48 (20.6)	185 (79.4)	<0.001	0.50 (0.37, 0.68)
	No	62 (41.1)	89 (58.9)		
Frequency of children’s visits to family					
No visits or dead children		21 (37.5)	35 (62.5)	0.113	1.38 (0.94, 2.02)
Visitation by children		89 (27.1)	239 (72.9)		
Smoking					
	Yes	12 (26.8)	115 (73.2)	0.495	1.04 (0.92, 1.18)
	No	68 (30.0)	159 (70.0)		
Alcohol consumption					
	Yes	90 (32.6)	186 (67.4)	<0.001	1.76 (1.14, 2.70)
	No	20 (18.5)	88 (81.5)		
Depression					
No or minimal depression		08 (7.1)	106 (92.9)	<0.001	1.49 (1.34, 1.66)
Depression		102 (37.8)	168 (62.2)		
Loneliness					
Low degree of loneliness		28 (14.4)	167 (85.6)	<0.001	1.51 (1.31, 1.73)
High degree of loneliness		82 (43.4)	107 (56.5)		

Higher levels of depressive symptoms were found in the following groups: ≥80 years of age (n = 71, 83.5%), females (n = 128, 77.1%), illiterate (n = 183, 82.4%), rural residents (n = 170, 88.5%), living alone (n = 130, 86.1%), those with no visits from their children or whose children died (n = 51, 91.1%), and alcohol users (n = 209, 75.7%). These associations were found to be statistically significant, as seen in Table 3.

**Table 3:** Factors associated with depression among the elderly (n = 384).

Factor			Minimal depression, n (%)	Some depression, n (%)	p-value	Unadjusted odds ratio (95% CI)
Age group						
<80 years and ≥80 years			100 (33.4)	199 (66.6)	0.003	2.03 (1.22, 3.36)
			14 (16.5)	71 (83.5)		
Gender						
Male, Female			76 (34.9)	142 (65.1)	0.011	1.80 (1.14, 2.85)
			38 (22.9)	128 (77.1)		
Educational status						
No formal education			39 (17.6)	183 (82.4)	<0.001	2.25 (1.16, 3.39)
Formal education			75 (46.3)	87 (53.7)		
Occupational status						
Currently not employed			6 (11.8)	45 (88.2)	0.003	0.28 (0.12, 0.67)
Currently employed			108 (32.4)	225 (67.6)		
Type of residence area						
Rural			22 (11.5)	170 (88.5)	<0.001	0.14 (0.08, 0.24)
Urban			92 (47.9)	100 (52.1)		
Living with spouse						
	Yes		93 (39.9)	140 (60.1)	<0.001	0.25 (0.15, 0.90)
	No		21 (13.9)	130 (86.1)		
Frequency of children’s visits to family						
No visits or dead children			5 (8.9)	51 (91.1)	<0.001	0.20 (0.08, 0.51)
Visitation by children			109 (33.2)	219 (66.8)		
Smoking status						
		Yes	38 (24.2)	119 (75.8)	0.050	2.63 (0.40, 3.52)
		No	76 (33.5)	151 (66.5)		
Alcohol consumption						
		Yes	67 (24.3)	209 (75.7)	<0.001	1.42 (1.26, 3.67)
		No	47 (43.5)	61 (56.5)		

Higher degrees of loneliness were found in the following groups: ≥80 years of age (n = 51, 60%), illiterate (n = 136, 61.3%), rural residence (n = 120, 62.5%), living alone (n = 106, 70.2%), those not visited by children or whose children were dead (n = 46, 82.1%), smokers (n = 89, 56.7%) and alcohol users (n = 149, 54%). These associations were found to be statistically significant, as seen in Table 4.

**Table 4:** Factors associated with loneliness among the elderly (n = 384).

Factor		Low degree of loneliness, n (%)	Moderate to severe degree of loneliness, n (%)	p-value	Unadjusted odds ratio (95% CI)
Age group					
<80 years and ≥80 years		161 (53.8)	138 (46.2)	0.024	1.75 (1.07, 2.85)
		34 (40)	51 (60)		
Gender					
	Male	115 (52.8)	103 (47.2)	0.376	1.20 (0.80, 1.80)
	Female	80 (48.2)	86 (51.8)		
Educational status					
No formal education		86 (38.7)	136 (61.3)	<0.001	2.31 (1.20, 4.47)
Formal education		109 (67.3)	53 (32.7)		
Occupational status					
Currently not employed		21 (41.2)	30 (58.8)	0.141	0.64 (0.35,1.16)
Currently employed		174 (52.3)	159 (47.7)		
Type of residence area					
Rural		72 (37.5)	120 (62.5)	<0.001	0.34 (0.22, 0.51)
Urban		123 (64.1)	69 (35.9)		
Living with spouse					
	Yes	150 (64.4)	83 (35.6)	<0.001	0.46 (0.14, 0.61)
	No	45 (29.8)	106 (70.2)		
Frequency of children’s visits to family					
No visits or dead children		10 (17.9)	46 (82.1)	<0.001	0.17 (0.08, 0.34)
Visitation by children		185 (56.4)	143 (43.6)		
Smoking status					
	Yes	68 (43.3)	89 (56.7)	0.015	2.60 (1.40, 4.91)
	No	127 (55.9)	100 (44.1)		
Alcohol consumption					
	Yes	127 (46)	149 (54)	0.003	1.50 (1.12, 3.80)
	No	68 (63)	40 (37)		

Multivariate logistic regression was done for factors found by the univariate analysis to be significantly associated with CI, depression, and loneliness. The results are presented in Table 5.

**Table 5:** Adjusted odds ratios for the factors associated with depression, loneliness, and cognitive impairment among the study participants (n = 384). * P < 0.001.

Variable	Adjusted odds ratio for loneliness among the elderly	Adjusted odds ratio for depression among the elderly	Adjusted odds ratio for cognitive impairment among elderly
Age > 80 years	2.82 (0.45, 3.82)	2.39 (1.11, 5.13)	2.82 (0.54, 4.85)
Gender	1.39 (0.66, 2.90)	0.90 (0.41, 1.98)	2.38 (0.82, 6.91)
Educational status	1.84 (1.05, 3.23)	1.29 (0.69, 2.43)	2.85 (1.35, 4.45)
Type of resident area	0.30 (0.17, 0.53)^*^	0.12 (0.06, 0.24)^*^	0.18 (0.04, 0.81)
Living with spouse	0.24 (0.14 0.41)^*^	0.26 (0.14, 0.50)^*^	0.23 (0.11, 0.78)
Smoking	1.86 (0.98, 3.54)	2.04 (1.02, 4.08)	0.57 (0.21, 1.59)
Alcohol consumption	1.14 (0.64, 2.03)	1.73 (0.92, 3.25)	0.35 (0.14, 0.87)
Depression	___________	___________	4.49 (1.37, 14.67)

## Discussion

In our study, we utilized the MOCA scale to assess CI among the study participants. We found that CI (in any form) had a prevalence of 91.7%, with 28.6% of participants exhibiting severe CI. Our study found a higher prevalence of CI compared to studies conducted elsewhere, which reported a prevalence ranging from 4.5% to 71.9% [15-23]. The higher prevalence in our study can be ascribed to the study group’s profile, with all participants ≥65 years of age, ranging from 65 to 95 years. Nonetheless, the higher prevalence highlights the importance of routine screening of those older than 65 years for various forms of CI using validated tools such as MOCA, which will help in early diagnosis and implementation of interventions, leading to improved quality of life among the elderly.

Illiteracy and depression were positively associated with higher forms of CI, whereas living in urban areas, living with spouses, and not consuming alcohol were found to be negatively associated with higher forms of CI. Illiteracy has been reported as an important risk factor associated with CI in studies conducted in multiple settings across the world [15-17,20,21,24,25]. The plausibility of illiteracy as a risk factor for CI can be explained by the fact that education is an important aspect of cognitive functioning. Therefore, a lack of education can affect the cognitive abilities of an individual. Similarly, depression has been reported as an important risk factor associated with CI in multiple studies [20,21,26,27]. The positive association between depression and CI may be attributed to the involvement of similar neurological and pathological mechanisms in the development of both conditions. The hypothesis that depression may be a prodromal symptom of CI should be explored further. Urban dwelling was found to be a protective factor against CI in our study. Previous studies have reported rural dwellings as a risk factor for CI [17,20,21,28-30]. These findings are in contrast with traditional wisdom in countries like India, where people living in rural societies are assumed to have a better social life, camaraderie with others, and a sense of community, which are thought to provide protection against a decline in cognition. However, our findings reveal the opposite effect, highlighting the importance of screening elders residing in rural India, which may help to identify the hidden burden of dementia in the country.

Living with a spouse was found to be a protective factor against CI in our study, which agreed with the findings of other studies [15,18,20,23,25,26,31]. Being alone in old age can be difficult, considering the occurrence of degenerative physical and psychological changes. However, living with a partner can help one cope with the stress occurring as a result of these changes. This finding suggests the importance of providing companionship to elderly individuals, whether among family or friends. Avoiding alcohol consumption was also found to be a protective factor against CI, as observed in other studies conducted elsewhere [17,19,26]. Alcohol is a known intoxicant associated with the development of neurodegenerative disorder. Chronic alcoholism is associated with various types of cognitive impairment not only among the elderly but also younger individuals. Community-based interventions to reduce alcohol dependency may be highly effective in reducing the burden of CI among the elderly.

Based on our univariate analysis, other factors that were found to be significantly associated with CI were >80 years of age, female gender, and higher degrees of loneliness. However, multivariate logistic regression analysis showed these associations were not statistically significant. Nonetheless, addressing these factors can help in the early identification of those who are at risk of developing CI.

In our study, we found that nearly half of the participants (49.2%) experienced moderate to high degrees of loneliness. Interestingly, higher degrees of loneliness were seen among those living in rural areas. These findings speak to the changing living patterns of those living in countries like India, where the proportion of the elderly population is gradually increasing over time. As India is primarily a country of villages, aging and its associated conditions are also likely to be experienced to a greater extent by those living in rural areas compared to the urban populace. Therefore, extensive geriatric health care should be included in the existing primary healthcare framework so that timely preventive intervention can be provided to those in need.

We also found that nearly half (39.3%) of the participants experienced moderate to severe depression. Higher degrees of depression were associated with various factors, including those >80 years of age, female gender, illiteracy, rural residence, living alone, those whose children did not visit or had died, and alcohol use. Depression in old age is a multifactorial occurrence, and the identification and addressing of key factors through timely interventions (e.g., proper counseling, guidance, and pharmacotherapy) will help the elderly cope with their depressive symptoms, indirectly improving their cognitive outcome.

Our study did have some limitations, including a smaller sample size compared to other studies. We also did not collect data on important clinical parameters, such as hypertension and diabetes mellitus. These limitations could limit the generalizability of our study. Nonetheless, our study also had some strengths, such as our comparison of urban and rural populations, the inclusion of loneliness and depression aspects, and the use of fully community-based research and logistic regression techniques for data analysis, which helped to identify important factors associated with cognitive impairment.

## Conclusions

Cognitive impairment is a serious public health problem in India, which is increasing as the country is developing further. Factors such as depression and loneliness are further compounding the problem by increasing the vulnerability of the elderly to various degrees of CI, which not only reduces quality of life but may also lead to injuries or accidents. The risk of mortality and long duration of confinement to bed makes living conditions poor for the elderly population.

For a population that is as diverse and large as that of India, the time has come to develop mechanisms (e.g., cognitive surveillance and a CI register) for the longitudinal assessment of the country’s CI burden. Timely interventions, such as early identification through community-based screening, the inclusion of a geriatric health component in primary health care, and proper counseling, will go a long way in addressing and reducing the burden of cognitive impairment in the community.
